# Quality ratings of reviews in overviews: a comparison of reviews with and without dual (co-)authorship

**DOI:** 10.1186/s13643-018-0722-9

**Published:** 2018-04-24

**Authors:** Dawid Pieper, Andreas Waltering, Jakob Holstiege, Roland Brian Büchter

**Affiliations:** 1 Institute for Research in Operative Medicine, Witten/Herdecke University, Ostmerheimer Str. 200 (Building 38), 51109 Cologne, Germany; 2 Institute for Quality and Efficiency in Health Care (IQWiG), Im Mediapark 8, 50670 Cologne, Germany; 3 Central Research Institute of Ambulatory Health Care in Germany (ZI), Herbert-Lewin-Platz 3, 10623 Berlin, Germany

**Keywords:** Conflict of interest, Overview, Systematic review, Methods

## Abstract

**Background:**

Previous research shows that many authors of Cochrane overviews were also involved in some of the included systematic reviews (SRs). This type of dual (co-)authorship (DCA) may be a conflict of interest and a potential source of bias. Our objectives were to (1) additionally investigate DCA in non-Cochrane overviews; (2) investigate whether there is an association between DCA and quality assessments of SRs in Cochrane and non-Cochrane overviews.

**Methods:**

We selected a sample of Cochrane (*n* = 20) and non-Cochrane (*n* = 78) overviews for analysis. We extracted data on the number of reviews affected by DCA and whether quality assessment of included reviews was conducted independently. Differences in mean quality scores between SRs with and without DCA were calculated in each overview. These differences were standardized (using the standardized mean difference (SMD)) and meta-analyzed using a random effects model.

**Results:**

Forty out of 78 non-Cochrane overviews (51%) and 18 out of 20 Cochrane overviews (90%) had included at least one SR with DCA. For Cochrane overviews, a median of 5 [interquartile range (IQR) 2.5 to 7] SRs were affected by DCA (median of included reviews 10). For non-Cochrane overviews a median of 1 [IQR 0 to 2] of the included SRs were affected (median of included reviews 14). The meta-analysis showed a SMD of 0.58 (95% confidence interval (CI) 0.27 to 0.90) indicating higher quality scores in reviews with overlapping authors. The test for subgroup differences shows no evidence of a difference between Cochrane (SMD 0.44; 95% CI 0.07 to 0.81) and non-Cochrane overviews (SMD 0.62; 95% CI 0.06 to 1.17).

**Conclusions:**

Many authors of overviews also often have an authorship on one or more of the underlying reviews. Our analysis shows that, on average, authors of overviews give higher quality ratings to SRs in which they were involved themselves than to other SRs. Conflict of interest is one explanation, but there are several others such as reviewer expertise. Independent and blinded reassessments of the reviews would provide more robust evidence on potential bias arising from DCA.

## Background

Overviews of systematic reviews (henceforth termed overviews) attempt to systematically retrieve, assess, and synthesize the results of multiple systematic reviews (SRs) for a given condition or public health problem [1]. The number of published overviews is rapidly increasing [2, 3].

“Systematic reviewers” has become a term for people conducting SRs. We would expect systematic reviewers to also be involved in the conduct of overviews. Thus, authors of overviews might include SRs into their overviews which they have (co-)authored. We employ the term dual (co-)authorship (DCA) to describe this scenario [4]. Such an overlap in authorship can be considered a competing interest and raises questions regarding conflicts of interests. In theory, several steps in the conduct of an overview may be biased by DCA such as formulating inclusion criteria, conducting quality assessments, interpreting data, drawing conclusions, or dealing with competing reviews. Experts in a given field might be more likely to participate in an overview, while being enthusiastic for specific interventions, or have strong views about its effectiveness, for instance. Their opinion might also be bias by financial conflicts of interest. For example, a recent analysis found review sponsorship and authors’ financial conflicts of interest to introduce bias affecting the outcomes of reviews that could not be explained by other sources of bias [5].

In a sample of 197 Cochrane reviews, 28 (14%) were affected by DCA. DCA was mentioned in 68% (19/28) of the cases as a potential conflict of interest [6]. Our former study found that most (90%) Cochrane overviews were affected by DCA (i.e., at least one of the included reviews was affected by DCA) [4]. In 9/18 (50%) Cochrane overviews with DCA, quality assessment was not conducted independently (i.e., at least one person who (co-)authored the review was involved in quality assessment). To the best of our knowledge, no such data are available for non-Cochrane overviews. Furthermore, our former analysis focused only on the prevalence and management of DCA.

In this study, our objectives are to (1) investigate DCA in non-Cochrane overviews; (2) investigate whether there is an association between DCA and quality assessments of SRs in Cochrane and non-Cochrane overviews.

## Methods

There was no a priori protocol for the study.

Given that our study had two objectives, the methods and results section are each separated into two parts. The first deals with the analysis of DCA in non-Cochrane overviews and a comparison with Cochrane overviews. The second describes a comparison of quality assessments of reviews with and without DCA using meta-analytical methods. The second part of the analysis comprises data of Cochrane and non-Cochrane reviews. The data on Cochrane overviews is taken from our former study [4].

### DCA in non-Cochrane overviews

To allow for the comparability of results, the methods for the analysis of non-Cochrane overviews followed the same methods as our former study on Cochrane overviews [4]. In brief, we searched MEDLINE(via Pubmed) with a precision-maximizing search strategy (overview[ti] AND reviews[ti]) for overviews published from 2010 to September 2015. Our definition of an overview followed criteria outlined as follows [7]:

1. Overviews should contain a clearly formulated objective designed to answer a specific clinical research question, typically about a healthcare intervention.
2. Overviews should intend to search for and include only systematic reviews (with or without meta-analyses).
3. Overviews should use explicit and reproducible methods to identify multiple systematic reviews that meet their inclusion criteria and to assess the methodological quality of these systematic reviews.
4. Overviews should intend to collect, analyze, and present the descriptive characteristics of their included systematic reviews (and their primary studies) and the quantitative outcome data contained within the systematic reviews.

Protocols were excluded. In cases where updates were published, we used the most recent version. Overview selection was performed applying liberal acceleration (i.e., all titles and abstracts were screened by one reviewer; those deemed not relevant were verified by a second person for exclusion). All data were extracted by one person and verified by a second person. Data were extracted on the same items as in our former study [4]. Data were analyzed descriptively as frequencies or medians and interquartile ranges (IQR). To compare Cochrane and non-Cochrane overviews we used the Mann-Whitney *U* statistic and calculated odds ratios with 95% confidence intervals.

### Comparison of quality assessments of reviews with and without DCA

We used meta-analytical methods to compare the quality assessments of the included SRs with versus without DCA. For this, we extracted data on quality assessments of the SRs from the overviews. The methodological quality of the included SRs was assessed in the overviews using various tools and was reported in different ways.

The Assessment of Multiple Systematic Reviews (AMSTAR) tool [8], R(evised)-AMSTAR [9] and the Overview Quality Assessment Questionnaire (OQAQ) [10] were frequently used to assess the methodological quality of SRs. AMSTAR consists of 11 items, each of which is categorized into a standardized set of four possible responses: “yes”, “no”, “cannot answer”, or “not applicable” [8]. The OQAQ was used in the development of AMSTAR. R-AMSTAR was developed to quantify the methodological quality by assigning a quality score to each SR ranging from 11 to 44, with higher scores indicating higher quality [9]. The OQAQ consists of 10 items, the first nine of which focus on methodological aspects of the scientific quality of a SR, while the last item provides an overall assessment based on an ordinal scale ranging from 1 to 7, with higher scores indicating less flaws (i.e., higher quality) [10]. The first nine questions each have three possible responses: “yes,” “no,” or “partial/cannot tell.”

For AMSTAR, a total score can be derived by summing up the number of “yes” items. This was done when authors did not present an overall score. Where overall scores were reported, these were extracted along with information on how a score was calculated to account for modifications of the original tool. In this regard, R-AMSTAR and the OQAQ were treated in the same way. In cases where authors applied or reported the results of the quality assessment on an ordinal scale (i.e., high, medium, low quality) we assigned numerical values, i.e., “high” was given a score of three, “medium” was given a score of 2, and low was given a score of 1, so that a higher value indicates a higher methodological quality. All data extractions were performed by one person and checked by a second for accuracy. Disagreements were resolved by discussion. We did not approach any authors for additional data.

Overviews had to meet the following criteria to be eligible for inclusion in the meta-analytical analysis:

- At least two reviews affected by DCA and two reviews not affected by DCA included (to allow for calculation of a SD)
- SD greater than 0 (i.e., the quality ratings varied across SRs)

Within each overview, we calculated the difference in mean quality score between SRs with and without DCA. These differences were standardized by the pooled SD. We conducted random effects meta-analyses (MAs) using DerSimonian and Laird’s heterogeneity variance estimator. All analyses were performed with RevMan 5.3. The included SRs served as units of analysis. The standardized mean difference (SMD) was chosen as the principal summary measure in meta-analysis to account for different scales. Mean differences (MD) were calculated when all overviews used the same scale. We used *I*^2^ to quantify inconsistency [11].

We expected skewed data due to unbalanced and small frequencies of reviews per group. Therefore, we checked the data by calculating the observed mean minus the lowest possible value (e.g., 1 for AMSTAR) and by dividing this by the SD [11]. A ratio less than 2 suggests skew, while there is strong evidence for a skewed distribution if the ratio is less than 1 [12]. We performed a sensitivity analysis by excluding all overviews where the ratio was less than 2 in any of the two groups, i.e., reviews with or without DCA. A subgroup analysis was also performed for Cochrane overviews and non-Cochrane overviews. Further meta-analyses were conducted for overviews using the original quality assessment instruments without any modifications. We were not able to investigate the impact of independent (i.e., quality assessment is performed by authors without DCA) versus non-independent quality assessment of SRs in overviews with overlapping authors due to (too) few overviews in this subsample.

## Results

### DCA in non-Cochrane overviews

In total, we included 78 non-Cochrane overviews (see Appendix for list of included and excluded overviews). They included a median of 14 reviews (interquartile range (IQR), 8.25–24). In 40 of 78 non-Cochrane overviews (51%), at least one of the included reviews was affected by DCA, and a median of 1 (IQR, 0–2) reviews per non-Cochrane overview were affected by DCA. In 8 out of these 40 overviews (20%), quality assessment was conducted independently. Two non-Cochrane overviews affected by DCA described this as a limitation, and four as a declaration of interest. Safeguards against potential bias arising from DCA were described in two non-Cochrane overviews. Table 1 illustrates this by contrasting these figures to the results of our former study on Cochrane overviews.

**Table 1:** Comparison of Cochrane overviews and non-Cochrane overviews

	Cochrane overviews (*n* = 20)	Non-Cochrane overviews (*n* = 78)	Mann-Whitney *U* test (*p* value)	Odds ratio (95% CI)
Included SRs (median, IQR)	10 (6; 18.5)	14 (8.25; 24)	0.184	
SRs affected by DCA per overview (median, IQR)	5 (2.5; 7)	1 (0; 2)	< 0.001	
Overviews affected by DCA	18 (90%)	40 (51%)*		8.6 (1.9–39.4)
Independent assessment	8 (44%)	8 (20%)		3.2 (1.0–10.7)
SRs not assessed independently (median, IQR)	0 (0; 4.25)	1 (0; 2)	0.787	
Reported as limitation	5 (28%)	2 (5%)		7.3 (1.3–42.3)
Reported as declaration of interest	11 (61%)	4 (10%)		11.0 (2.8–42.8)
Safeguard	7 (39%)	2 (5%)		12.1 (2.2–66.8)

*CI* confidence interval, *SRs* systematic reviews, *IQR* interquartile range, *DCA* dual (co-)authorship

*Two overviews were assessed as partly and are not included in the denominator

### Results from meta-analytical comparison

**Fig. 1:**
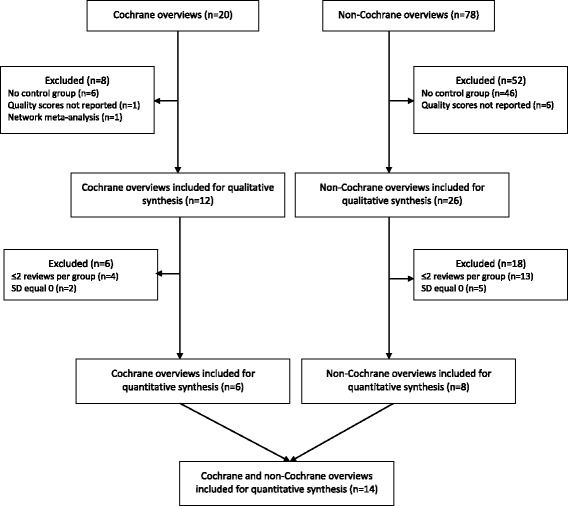
Flow Chart

Out of 20 Cochrane overviews and 78 non-Cochrane overviews included in the descriptive analysis, 14 overviews (6 Cochrane overviews and 8 non-Cochrane overviews) were included in the meta-analysis (see Fig. 1). All Cochrane overviews applied AMSTAR to assess the methodological quality of the included SRs. Four of these applied the original instrument [13–16], while one of them calculated a percentage score to account for “not applicable” responses [16]. Two Cochrane overviews modified AMSTAR allowing for a maximum score of 10 [17, 18]. Among non-Cochrane overviews, three applied the original AMSTAR version [19–21], while five applied the original OQAQ version [22–26].

**Fig. 2:**
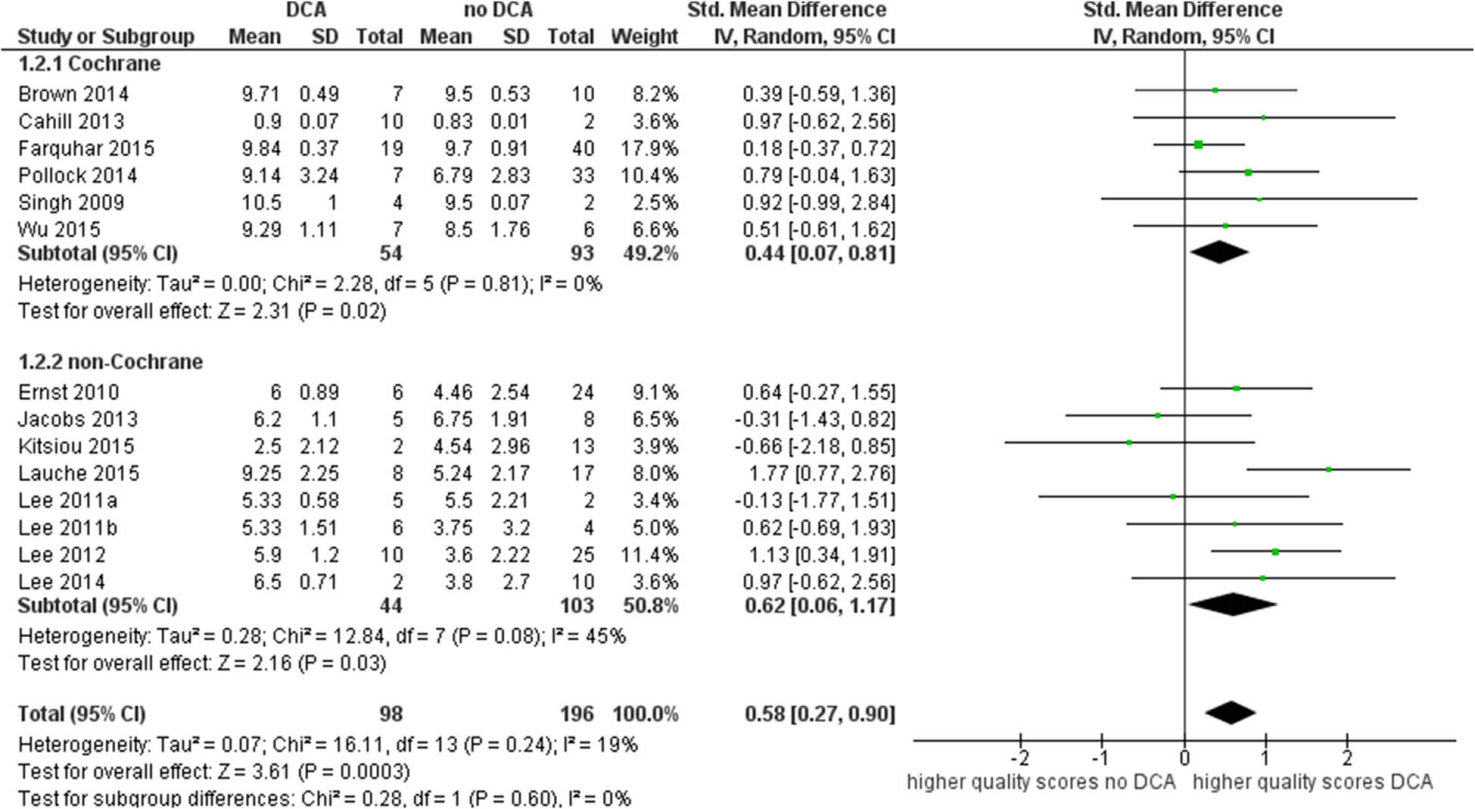
Mean quality scores

In all but three overviews mean quality scores were higher for reviews with DCA. The meta-analysis showed a SMD of 0.58 (95% confidence interval (CI) 0.27 to 0.90) indicating higher quality scores in reviews with overlapping authors (see Fig. 2). There was little inconsistency in the observed SMDs (*I*^2^ = 19%, *p* = 0.24). The test for subgroup differences shows no evidence of a difference between Cochrane (SMD 0.44; 95% CI 0.07 to 0.81) and non-Cochrane overviews (SMD 0.62; 95% CI 0.06 to 1.17). The difference in subgroup estimates was 0.18 (95% CI − 0.48 to 0,84, *p* value 0.60) as calculated by *Z* test. There was some evidence of inconsistency in the meta-analysis for non-Cochrane overviews (*I*^2^ = 45%, *p* = 0.08), while no inconsistency was observed for Cochrane overviews (*I*^2^ = 0%, *p* = 0.60).

**Fig. 3:**
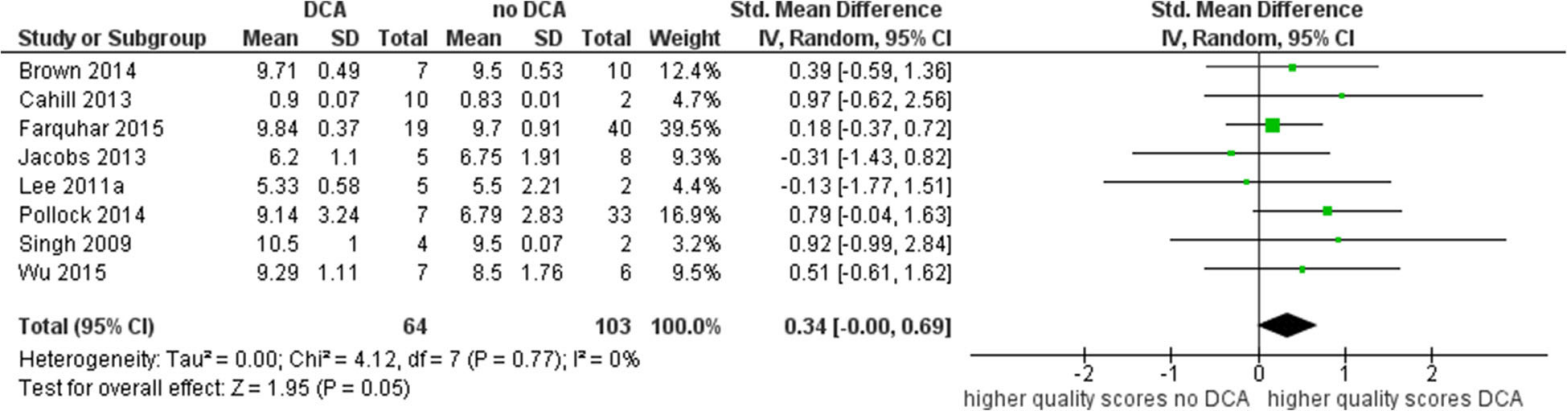
Mean quality scores (sensitivity analysis)

Six overviews were excluded due to skew data in the sensitivity analysis (see Fig. 3). All six excluded overviews were non-Cochrane overviews. Thus, the sensitivity analysis resembles the subgroup analysis for Cochrane overviews. However, the effect decreased to a SMD of 0.34 (95% CI − 0.00 to 0.69), with no evidence of inconsistency (*I*^2^ = 0%, *p* = 0.77).

**Fig. 4:**
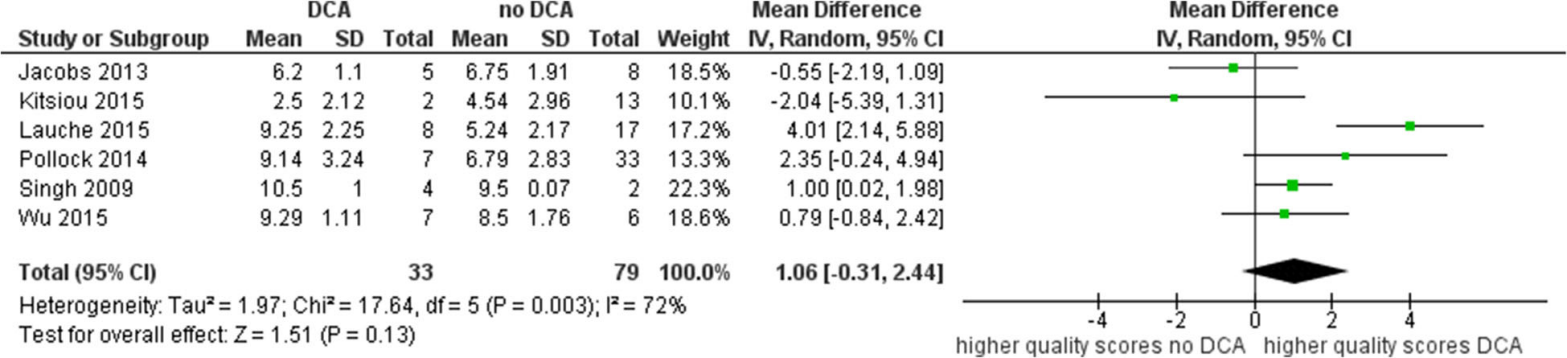
Mean quality scores for AMSTAR

**Fig. 5:**
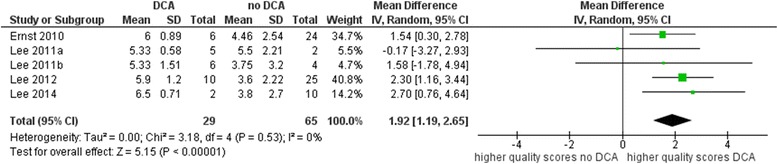
Mean quality scores for OQAQ

In total, six overviews used the original AMSTAR version without any modifications. The meta-analysis showed that reviews affected by DCA were scored one point higher than reviews not affected by DCA with respect to their methodological quality. The MD was 1.06 (95% CI − 0.31 to 2.44), with strong evidence of inconsistency (*I*^2^ = 72%, *p* = 0.003) (see Fig. 4). The effect was stronger for the OQAQ. The MD was 1.92 (95% CI 1.19 to 2.65), with no evidence of inconsistency (*I*^2^ = 0%, *p* = 0.53) based on five overviews (see Fig. 5).

## Discussion

Within this study, we compared DCA of Cochrane and non-Cochrane overviews and investigated whether there is an association between DCA and quality assessments of SRs in Cochrane and non-Cochrane overviews.

The comparison of Cochrane overviews with non-Cochrane overviews revealed significant differences with respect to the prevalence of DCA. While nearly all Cochrane overview were affected by DCA to some extent, this was the case for only half of non-Cochrane overviews (90 vs 51%). Furthermore, the proportion of reviews affected by DCA was much larger in Cochrane overviews. Since the Cochrane Collaboration is dedicated to evidence syntheses, we would expect clustering of authors in overviews. The higher proportion of overlaps in Cochrane overviews can be explained by the fact that Cochrane overviews usually exclude non-Cochrane reviews [4].

However, authors of Cochrane overviews were also more aware of the problems that might arise from dual (co-)authorship. They more often considered DCA to be a limitation or reported it in the declaration of interest section. Also, quality assessments of included reviews with DCA were more often conducted by authors not involved in the reviews. This may be due to higher awareness of conflict of interests among Cochrane reviewers or Cochrane policies. Both the declarations of interests section as well as Cochrane’s code of conduct in the Cochrane Handbook emphasize independence, transparency and acknowledgement of conflicts of interest [27]. Furthermore, minimizing bias by avoiding conflicts of interest is also stated as a goal in the fourth principle of the Cochrane Collaboration [28]. Specifically, the Cochrane policy stipulates that authors should not extract data from or assess quality of research they were involved in. Such stringent policies do not seem to exist for authors conducting overviews outside of the Cochrane Collaboration or other similarly spirited organizations. A recent survey of SRs showed that statements on conflicts of interest are more often (100 vs. 83%) included in Cochrane reviews than in non-Cochrane reviews [29]. In another survey, 97% of SRs reported conflict of interest disclosures [30]. In this study, which specifically looked at non-financial conflicts of interest, Cochrane authors more frequently reported such conflicts of interest compared to non-Cochrane authors (19 vs. 5%, *p* = 0.004) [30].

While the majority of medical journals require conflict of interest statements nowadays, only about half require statements on non-financial conflicts of interest and hardly any ask for intellectual conflicts of interest specifically [31], although conflicts of interest definitions often vary [32]. Intellectual conflicts of interest are defined as “academic activities that create the potential for an attachment to a specific point of view that could unduly affect an individual’s judgment about a specific recommendation” [33]. However, there is still a discussion in the scientific community about the presence of intellectual conflicts of interest [34–36].

Despite the conflict of interest policies within the Cochrane Collaboration, our study showed that reviews affected by DCA obtained higher quality scores than reviews not affected DCA in Cochrane overviews. This finding also occurred in non-Cochrane overviews. Overviews with DCA scored one and two points higher in overviews applying the original AMSTAR or OQAQ tool, respectively. When interpreting this, it is important to keep in mind that the range of possible scores is 0–11 and 1–7 for AMSTAR and OQAQ, respectively. Thus, the difference of two points for the OQAQ is also more important in relative terms as the scale is shorter than for AMSTAR. A possible explanation for the difference between both tools observed here is the subjectivity of the OQAQ. However, there is no guidance on how to derive at the overall assessment. Counting the number of “yes” items in AMSTAR is therefore to a lesser extent subjective. It is also important to keep in mind that deriving an overall score is inherent in application of the OQAQ. A sum score is not mentioned for AMSTAR in its source publication and has never been validated [8]. It can be questioned whether any of these differences are relevant in terms of interpreting the methodological quality of a SR. Generally, a 1-point difference in AMSTAR should not reflect huge differences of methodological quality between SRs, although this might depend on the item affected by the judgment. For example, application of unjustified statistical methods will usually have a higher impact on the methodological quality of a SR than not providing a list of included and excluded studies. Nevertheless, it should be kept in mind that it is also common to categorize SRs based on their AMSTAR score. For example, the Canadian Agency for Drugs and Technologies in Health (CADTH) determines categories of quality as follows: low (score 0 to 3), medium (score 4 to 7), and high (score 8 to 11) [37]. When a cut-off is used as an inclusion or exclusion criterion for a SR within in overview, a 1-point difference may have an important impact.

This is the first study empirically assessing dual (co-)authorship in overviews. Although our results show that reviews affected by DCA obtain higher scores for methodological quality than reviews not affected by DCA, the difference is not necessarily a result of authors’ biased quality assessments. Our analysis was performed at the overview level, and we did not collect any content-specific characteristics from the included reviews. Several other aspects might also explain the results. It is well established in the literature that Cochrane reviews have a higher methodological quality than non-Cochrane reviews [9, 38, 39]. Unfortunately, we were not able to include this in our analysis due to the low number of reviews included in overviews. In addition, the methodological quality of SRs has risen over time [40, 41]. This might be of interest when comparing several health care interventions in an overview where some interventions are more up-to-date than others. Also, the comparison of health care interventions from different fields (e.g., pharmacology, surgery, complementary alternative medicine) might be important as the quality of SRs is not necessarily equal among disciplines. All these potential explanatory variables might have an impact on the results of our analysis if they are not equally distributed among reviews with and without DCA.

We are not able to draw any definite conclusions based on our findings. For example, we were not able to investigate the impact of independent quality assessment of SRs (i.e., quality assessment is performed by independent authors) in overviews with DCA due to a too low number of overviews. Thus, we were only able to differentiate between reviews with and without DCA. While doing so, we assumed that overview authors that have co-authored an included SR pose a potential conflict of interest to the whole overview, irrespective of their tasks performed. It has been stressed that although quality assessment of a review is not performed by any of its own authors, evaluating a review of one of the other group members might also introduce bias [19]. Another idea would be to ask independent authors to evaluate the quality of SRs in overviews. However, this approach seems not feasible as the actual authors will already have a guess about the methodological quality after having performed study selection and data extraction. Furthermore, authors may find it difficult to draw conclusions when they did not perform assessments themselves. Authors of overviews and SRs may also be very well aware of the advantages and drawbacks of quality assessment tools causing them to report what is necessary in order to receive as much points as possible on a quality assessment scale (e.g., AMSTAR or OQAQ). Therefore, our results could be explained by differences in reporting rather than methodological quality.

We encourage future authors of SRs and overviews to report who was involved in which steps of study selection, data collection, and quality assessment by providing initials of persons performing these steps. This would allow further analyses in the future as this would allow differentiating between overviews where authors affected by DCA were involved in quality assessment, for example. In other words, while the unit of analysis is the SR in the current analysis, it could be shifted to single authors.

Future studies are needed to investigate the influence of reviews affected by DCA and to identify ways of how to best deal with it. Also, it would be prudent to investigate whether overviews not affected by DCA have a lower methodological quality. This could be done by a reassessment and comparison of the methodological quality of included reviews. Assessors should be blinded against the aim of the study, and their assessments would be compared with the original ratings. In the absence of bias (i.e., reviews affected by DCA in fact do have a higher methodological quality), both assessments should be comparable in theory. If bias is present, we would assume that scores of reviews affected by DCA would be lower in the reassessment than their original ratings, while no such effect would be observed in reviews without DCA. Needless to say, the issue of DCA is not overview specific, but also arises with systematic reviews and primary studies.

### Limitations

This study has some limitations which must be pointed out. First, our search strategy for the identification of non-Cochrane overviews followed a precision maximizing approach, thus our sample might lack representativeness. Second, we did not perform a sample size calculation before the study as sample sizes calculations are known to be difficult in this context [42]. Third, the analyzed data is skewed and unbalanced. This might question the use of standard methods for meta-analyses of parametric data. However, we have tried to estimate impact of skew data by excluding them in a sensitivity analysis. Fourth, our study is based on quality scores of reviews. We have also calculated scores in cases where the authors refrained from doing this. This is true for AMSTAR where the overall score has never been validated and might be an inappropriate measure of methodological quality. However, there is no alternative approach we could have opt for to investigate differences in quality scores of reviews with and without DCA. Lastly, four of the included non-Cochrane overviews were published by one group of authors [23–26]. Thus, the results of these overviews cannot be seen completely independent of each other.

## Conclusions

DCA frequently occurs in overviews. Nearly all Cochrane overviews are affected by DCA. Reviews with DCA obtain higher methodological quality scores than reviews without. Potential conflicts of interest are one explanation for this association. The reasons need to be further investigated, however. Authors need guidance what to do if they are going to include their own review.

## Appendix

### Descriptive analysis

#### Included Cochrane overviews

Anderson L, Taylor RS. Cardiac rehabilitation for people with heart disease: an overview of Cochrane systematic reviews. Cochrane Database of Systematic Reviews 2014, Issue 12.

Brown J, Farquhar C. Endometriosis: an overview of Cochrane Reviews. Cochrane Database of Systematic Reviews 2014, Issue 3.

Cahill K, Stevens S, Perera R, Lancaster T. Pharmacological interventions for smoking cessation: an overview and network meta-analysis. Cochrane Database of Systematic Reviews 2013, Issue 5.

Cates CJ, Oleszczuk M, Stovold E, Wieland LS. Safety of regular formoterol or salmeterol in children with asthma: an overview of Cochrane reviews. Cochrane Database of Systematic Reviews 2012, Issue 10.

Cates CJ, Wieland LS, Oleszczuk M, Kew KM. Safety of regular formoterol or salmeterol in adults with asthma: an overview of Cochrane reviews. Cochrane Database of Systematic Reviews 2014, Issue 2.

Derry CJ, Derry S, Moore RA. Sumatriptan (all routes of administration) for acute migraine attacks in adults - overview of Cochrane reviews. Cochrane Database of Systematic Reviews 2014, Issue 5.

Farquhar C, Rishworth JR, Brown J, Nelen WLDM, Marjoribanks J. Assisted reproductive technology: an overview of Cochrane Reviews. Cochrane Database of Systematic Reviews 2014, Issue 12.

Flodgren G, Eccles MP, Shepperd S, Scott A, Parmelli E, Beyer FR. An overview of reviews evaluating the effectiveness of financial incentives in changing healthcare professional behaviours and patient outcomes. Cochrane Database of Systematic Reviews 2011, Issue 7.

Guay J, Choi P, Suresh S, Albert N, Kopp S, Pace NL. Neuraxial blockade for the prevention of postoperative mortality and major morbidity: an overview of Cochrane systematic reviews. Cochrane Database of Systematic Reviews 2014, Issue 1.

Hindocha A, Beere L, Dias S, Watson A, Ahmad G. Adhesion prevention agents for gynaecological surgery: an overview of Cochrane reviews. Cochrane Database of Systematic Reviews 2015, Issue 1.

Jones L, Othman M, Dowswell T, Alfirevic Z, Gates S, Newburn M, Jordan S, Lavender T, Neilson JP. Pain management for women in labour: an overview of systematic reviews. Cochrane Database of Systematic Reviews 2012, Issue 3.

Keus F, Gooszen HG, van Laarhoven CJHM. Open, small-incision, or laparoscopic cholecystectomy for patients with symptomatic cholecystolithiasis. An overview of Cochrane Hepato-Biliary Group reviews. Cochrane Database of Systematic Reviews 2010, Issue 1.

O’Connell NE, Wand BM, McAuley J, Marston L, Moseley GL. Interventions for treating pain and disability in adults with complex regional pain syndrome- an overview of systematic reviews. Cochrane Database of Systematic Reviews 2013, Issue 4.

Pollock A, Farmer SE, Brady MC, Langhorne P, Mead GE, Mehrholz J, van Wijck F. Interventions for improving upper limb function after stroke. Cochrane Database of Systematic Reviews 2014, Issue 11.

Ryan R, Santesso N, Lowe D, Hill S, Grimshaw J, Prictor M, Kaufman C, Cowie G, Taylor M. Interventions to improve safe and effective medicines use by consumers: an overview of systematic reviews. Cochrane Database of Systematic Reviews 2014, Issue 4.

Singh JA, Christensen R, Wells GA, Suarez-Almazor ME, Buchbinder R, Lopez-Olivo MA, Tanjong Ghogomu E, Tugwell P. Biologics for rheumatoid arthritis: an overview of Cochrane reviews. Cochrane Database of Systematic Reviews 2009, Issue 4.

Singh JA, Wells GA, Christensen R, Tanjong Ghogomu E, Maxwell LJ, MacDonald JK, Filippini G, Skoetz N, Francis DK, Lopes LC, Guyatt GH, Schmitt J, La Mantia L, Weberschock T, Roos JF, Siebert H, Hershan S, Cameron C, Lunn MPT, Tugwell P, Buchbinder R. Adverse effects of biologics: a network meta-analysis and Cochrane overview. Cochrane Database of Systematic Reviews 2011, Issue 2.

Welsh EJ, Evans DJ, Fowler SJ, Spencer S. Interventions for bronchiectasis: an overview of Cochrane systematic reviews. Cochrane Database of Systematic Reviews 2015, Issue 7. Art.

Wiffen PJ, Derry S, Moore RA, Aldington D, Cole P, Rice ASC, Lunn MPT, Hamunen K, Haanpaa M, Kalso EA. Antiepileptic drugs for neuropathic pain and fibromyalgia—an overview of Cochrane reviews. Cochrane Database of Systematic Reviews 2013, Issue 11.

Wu L, Norman G, Dumville JC, O’Meara S, Bell-Syer SEM. Dressings for treating foot ulcers in people with diabetes: an overview of systematic reviews. Cochrane Database of Systematic Reviews 2015, Issue 7.

#### Included non-Cochrane overviews

Adams, L.V., et al., Interventions to improve delivery of isoniazid preventive therapy: an overview of systematic reviews. BMC Infect Dis, 2014. 14: p. 281.

Andersen, J.H., et al., Risk factors for neck and upper extremity disorders among computers users and the effect of interventions: an overview of systematic reviews. PLoS One, 2011. 6(5): p. e19691.

Bao, Y., et al., Complementary and alternative medicine for cancer pain: an overview of systematic reviews. Evid Based Complement Alternat Med, 2014. 2014: p. 170396.

Berkhof, M., et al., Effective training strategies for teaching communication skills to physicians: an overview of systematic reviews. Patient Educ Couns, 2011. 84(2): p. 152–62.

Boaz, A., et al., Effective implementation of research into practice: an overview of systematic reviews of the health literature. BMC Res Notes, 2011. 4: p. 212.

Chan, R.J., E. Larsen, and *P. Chan*, Re-examining the evidence in radiation dermatitis management literature: an overview and a critical appraisal of systematic reviews. Int J Radiat Oncol Biol Phys, 2012. 84(3): p. e357–62.

Cheung, A., et al., Overview of systematic reviews of the effectiveness of reminders in improving healthcare professional behavior. Syst Rev., 2012. 1: p. 36.

Daka, Q. and V. Trkulja, Efficacy and tolerability of mono-compound topical treatments for reduction of intraocular pressure in patients with primary open angle glaucoma or ocular hypertension: an overview of reviews. Croat Med J, 2014. 55(5): p. 468–80.

Ernst, E. and M.S. Lee, Acupressure: an overview of systematic reviews. J Pain Symptom Manage, 2010. 40(4): p. e3–7.

Ernst, E. and M.S. Lee, Acupuncture for rheumatic conditions: an overview of systematic reviews. Rheumatology (Oxford), 2010. 49(10): p. 1957–61.

Ernst, E., M.S. Lee, and T.Y. Choi, Acupuncture in obstetrics and gynecology: an overview of systematic reviews. Am J Chin Med, 2011. 39(3): p. 423–31.

Ernst, E., M.S. Lee, and T.Y. Choi, Acupuncture for insomnia? An overview of systematic reviews. Eur J Gen Pract, 2011. 17(2): p. 116–23.

Ernst, E. and P. Posadzki, Complementary and alternative medicine for rheumatoid arthritis and osteoarthritis: an overview of systematic reviews. Curr Pain Headache Rep, 2011. 15(6): p. 431–7.

Ernst, E., P. Posadzki, and M.S. Lee, Complementary and alternative medicine (CAM) for sexual dysfunction and erectile dysfunction in older men and women: an overview of systematic reviews. Maturitas, 2011. 70(1): p. 37–41.

Faggion, C.M., Jr., et al., An overview of systematic reviews of the use of systemic antimicrobials for the treatment of periodontitis. Br Dent J, 2014. 217(8): p. 443–51.

Foisy, M., et al., Overview of Reviews The prevention of eczema in infants and children: an overview of Cochrane and non-Cochrane reviews. Evid Based Child Health, 2011. 6(5): p. 1322–1339.

Galvao, T.F., et al., Statins for early stage chronic kidney disease: an overview of reviews. Cardiovasc Hematol Disord Drug Targets, 2014. 14(3): p. 205–11.

Gamble, J.M., et al., Incretin-based medications for type 2 diabetes: an overview of reviews. Diabetes Obes Metab, 2015. 17(7): p. 649–58.

Gotink, R.A., et al., Standardized mindfulness-based interventions in healthcare: an overview of systematic reviews and meta-analyses of RCTs. PLoS One, 2015. 10(4): p. e0124344.

Hagen, K.B., et al., Exercise therapy for bone and muscle health: an overview of systematic reviews. BMC Med, 2012. 10: p. 167.

Heighes, P.T., et al., An overview of evidence from systematic reviews evaluating early enteral nutrition in critically ill patients: more convincing evidence is needed. Anaesth Intensive Care, 2010. 38(1): p. 167–74.

Huguet, A., et al., Efficacy of psychological treatment for headaches: an overview of systematic reviews and analysis of potential modifiers of treatment efficacy. Clin J Pain, 2014. 30(4): p. 353–69.

Hunt, K. and E. Ernst, The evidence-base for complementary medicine in children: a critical overview of systematic reviews. Arch Dis Child, 2011. 96(8): p. 769–76.

Iravani, M., et al., An overview of systematic reviews of normal labor and delivery management. Iran J Nurs Midwifery Res, 2015. 20(3): p. 293–303.

Jacobs, W.C., et al., The evidence on surgical interventions for low back disorders, an overview of systematic reviews. Eur Spine J, 2013. 22(9): p. 1936–49.

Johal, A., et al., Mandibular advancement splint (MAS) therapy for obstructive sleep apnoea-an overview and quality assessment of systematic reviews. Sleep Breath, 2015. 19(3): p. 1101–8.

Kang, H.S., et al., The use of acupuncture for managing gynaecologic conditions: An overview of systematic reviews. Maturitas, 2011. 68(4): p. 346–54.

Kansagara, D., et al., Transitions of Care from Hospital to Home: An Overview of Systematic Reviews and Recommendations for Improving Transitional Care in the Veterans Health Administration. 2015, Washington DC.

Kim, T.H., et al., Dietary supplements for benign prostatic hyperplasia: an overview of systematic reviews. Maturitas, 2012. 73(3): p. 180–5.

Kitsiou, S., G. Pare, and M. Jaana, Effects of home telemonitoring interventions on patients with chronic heart failure: an overview of systematic reviews. J Med Internet Res, 2015. 17(3): p. e63.

Kumar, A., S. Galeb, and B. Djulbegovic, Treatment of patients with multiple myeloma: an overview of systematic reviews. Acta Haematol, 2011. 125(1–2): p. 8–22.

Lauche, R., et al., A Systematic Overview of Reviews for Complementary and Alternative Therapies in the Treatment of the Fibromyalgia Syndrome. Evid Based Complement Alternat Med, 2015. 2015: p. 610615.

Laver, K., et al., Organising health care services for people with an acquired brain injury: an overview of systematic reviews and randomised controlled trials. BMC Health Serv Res, 2014. 14: p. 397.

Lee, M.S., et al., Aromatherapy for health care: an overview of systematic reviews. Maturitas, 2012. 71(3): p. 257–60.

Lee, M.S. and E. Ernst, Systematic reviews of t’ai chi: an overview. Br J Sports Med, 2012. 46(10): p. 713–8.

Lee, M.S. and E. Ernst, Acupuncture for surgical conditions: an overview of systematic reviews. Int J Clin Pract, 2014. 68(6): p. 783–9.

Lee, M.S., J.W. Kang, and E. Ernst, Does moxibustion work? An overview of systematic reviews. BMC Res Notes, 2010. 3: p. 284.

Lee, M.S., J.I. Kim, and E. Ernst, Is cupping an effective treatment? An overview of systematic reviews. J Acupunct Meridian Stud, 2011. 4(1): p. 1–4.

Lee, M.S., B. Oh, and E. Ernst, Qigong for healthcare: an overview of systematic reviews. JRSM Short Rep, 2011. 2(2): p. 7.

Lisy, K., H. White, and A. Pearson, Overview of reviews: mechanical interventions for the treatment and management of chronic obstructive pulmonary disease. Int J Nurs Pract, 2014. 20(6): p. 701–8.

Liu, L., et al., Acupuncture for low back pain: an overview of systematic reviews. Evid Based Complement Alternat Med, 2015. 2015: p. 328196.

Lockwood, C. and C. Stern, Interventions for the treatment of trachoma: an overview of Cochrane systematic reviews. Int J Nurs Pract, 2014. 20(6): p. 709–21.

Lodge, C.J., et al., Overview of evidence in prevention and aetiology of food allergy: a review of systematic reviews. Int J Environ Res Public Health, 2013. 10(11): p. 5781–806.

Long, L., et al., What is the clinical effectiveness and cost-effectiveness of conservative interventions for tendinopathy? An overview of systematic reviews of clinical effectiveness and systematic review of economic evaluations. Health Technol Assess, 2015. 19(8): p. 1–134.

Lu, L.Y., G.Q. Zheng, and Y. Wang, An overview of systematic reviews of shenmai injection for healthcare. Evid Based Complement Alternat Med, 2014. 2014: p. 840650.

Luo, J., et al., Traditional Chinese medicine injection for angina pectoris: an overview of systematic reviews. Am J Chin Med, 2014. 42(1): p. 37–59.

Luo, J., et al., Compound Danshen (Salvia miltiorrhiza) dripping pill for coronary heart disease: an overview of systematic reviews. Am J Chin Med, 2015. 43(1): p. 25–43.

Luo, J. and H. Xu, Outcome measures of chinese herbal medicine for coronary heart disease: an overview of systematic reviews. Evid Based Complement Alternat Med, 2012. 2012: p. 927392.

Luo, J., et al., Oral Chinese proprietary medicine for angina pectoris: an overview of systematic reviews/meta-analyses. Complement Ther Med, 2014. 22(4): p. 787–800.

Martineau, F., et al., Population-level interventions to reduce alcohol-related harm: an overview of systematic reviews. Prev Med, 2013. 57(4): p. 278–96.

Mbuagbaw, L., et al., Mobile phone text messaging interventions for HIV and other chronic diseases: an overview of systematic reviews and framework for evidence transfer. BMC Health Serv Res, 2015. 15: p. 33.

McCall, M.C., et al., Overview of systematic reviews: yoga as a therapeutic intervention for adults with acute and chronic health conditions. Evid Based Complement Alternat Med, 2013. 2013: p. 945895.

Misfeldt, R., et al., Incentives for improving human resource outcomes in health care: overview of reviews. J Health Serv Res Policy, 2014. 19(1): p. 52–61.

Momsen, A.M., et al., Multidisciplinary team care in rehabilitation: an overview of reviews. J Rehabil Med, 2012. 44(11): p. 901–12.

Patel, N.N. and G.D. Angelini, Pharmacological strategies for the prevention of acute kidney injury following cardiac surgery: an overview of systematic reviews. Curr Pharm Des, 2014. 20(34): p. 5484–8.

Plaszewski, M. and J. Bettany-Saltikov, Non-surgical interventions for adolescents with idiopathic scoliosis: an overview of systematic reviews. PLoS One, 2014. 9(10): p. e110254.

Posadzki, P., Is spinal manipulation effective for pain? An overview of systematic reviews. Pain Med, 2012. 13(6): p. 754–61.

Posadzki, P., L. Watson, and E. Ernst, Herb-drug interactions: an overview of systematic reviews. Br J Clin Pharmacol, 2013. 75(3): p. 603–18.

Posadzki, P., L.K. Watson, and E. Ernst, Adverse effects of herbal medicines: an overview of systematic reviews. Clin Med, 2013. 13(1): p. 7–12.

Reif, K., U. de Vries, and F. Petermann, [What does really help against cancer-related fatigue? An overview of systematic reviews]. Pflege, 2012. 25(6): p. 439–57.

Riech, A. and A. Schafer, [Standing- and gait therapy in adult patients after stroke: overview of reviews]. Rehabilitation (Stuttg), 2014. 53(6): p. 402–7.

Rotta, I., et al., Effectiveness of clinical pharmacy services: an overview of systematic reviews (2000–2010). Int J Clin Pharm, 2015.

Ryan, S.E., An overview of systematic reviews of adaptive seating interventions for children with cerebral palsy: where do we go from here? Disabil Rehabil Assist Technol, 2012. 7(2): p. 104–11.

Sutcliffe, P., et al., Aspirin for prophylactic use in the primary prevention of cardiovascular disease and cancer: a systematic review and overview of reviews. Health Technol Assess, 2013. 17(43): p. 1–253.

Terry, R., R. Perry, and E. Ernst, An overview of systematic reviews of complementary and alternative medicine for fibromyalgia. Clin Rheumatol, 2012. 31(1): p. 55–66.

Towler, P., A. Molassiotis, and S.G. Brearley, What is the evidence for the use of acupuncture as an intervention for symptom management in cancer supportive and palliative care: an integrative overview of reviews. Support Care Cancer, 2013. 21(10): p. 2913–23.

Tricco, A.C., et al., Seeking effective interventions to treat complex wounds: an overview of systematic reviews. BMC Med, 2015. 13: p. 89.

Wang, J. and X. Xiong, Outcome measures of chinese herbal medicine for hypertension: an overview of systematic reviews. Evid Based Complement Alternat Med, 2012. 2012: p. 697237.

Wei, X., et al., Complementary and Alternative Medicine for the Management of Cervical Radiculopathy: An Overview of Systematic Reviews. Evid Based Complement Alternat Med, 2015. 2015: p. 793649.

Wilson, M.G., et al., Counselling, case management and health promotion for people living with HIV/AIDS: an overview of systematic reviews. AIDS Behav, 2013. 17(5): p. 1612–25.

Worswick, J., et al., Improving quality of care for persons with diabetes: an overview of systematic reviews - what does the evidence tell us? Syst Rev., 2013. 2: p. 26.

Yang, C., et al., Efficacy and safety of acupuncture in children: an overview of systematic reviews. Pediatr Res, 2015. 78(2): p. 112–9.

Young, B., et al., Preventing childhood falls within the home: overview of systematic reviews and a systematic review of primary studies. Accid Anal Prev, 2013. 60: p. 158–71.

Young, T., et al., What are the effects of teaching evidence-based health care (EBHC)? Overview of systematic reviews. PLoS One, 2014. 9(1): p. e86706.

Yuan, J., et al., The efficacy and safety of alpha-1 blockers for benign prostatic hyperplasia: an overview of 15 systematic reviews. Curr Med Res Opin, 2013. 29(3): p. 279–87.

Zhang, J.H., D. Wang, and M. Liu, Overview of systematic reviews and meta-analyses of acupuncture for stroke. Neuroepidemiology, 2014. 42(1): p. 50–8.

Zou, K., et al., Preventing childhood scalds within the home: Overview of systematic reviews and a systematic review of primary studies. Burns, 2015. 41(5): p. 907–24.

Zwicker, J.G. and T.A. Mayson, Effectiveness of treadmill training in children with motor impairments: an overview of systematic reviews. Pediatr Phys Ther, 2010. 22(4): p. 361–77.

### Meta-epidemiological study

#### Included Cochrane overviews

Brown J, Farquhar C. Endometriosis: an overview of Cochrane Reviews. Cochrane Database of Systematic Reviews 2014, Issue 3.

Cahill K, Stevens S, Perera R, Lancaster T. Pharmacological interventions for smoking cessation: an overview and network meta-analysis. Cochrane Database of Systematic Reviews 2013, Issue 5.

Farquhar C, Rishworth JR, Brown J, Nelen WLDM, Marjoribanks J. Assisted reproductive technology: an overview of Cochrane Reviews. Cochrane Database of Systematic Reviews 2014, Issue 12.

Pollock A, Farmer SE, Brady MC, Langhorne P, Mead GE, Mehrholz J, van Wijck F. Interventions for improving upper limb function after stroke. Cochrane Database of Systematic Reviews 2014, Issue 11.

Singh JA, Christensen R, Wells GA, Suarez-Almazor ME, Buchbinder R, Lopez-Olivo MA, Tanjong Ghogomu E, Tugwell P. Biologics for rheumatoid arthritis: an overview of Cochrane reviews. Cochrane Database of Systematic Reviews 2009, Issue 4.

Wu L, Norman G, Dumville JC, O’Meara S, Bell-Syer SEM. Dressings for treating foot ulcers in people with diabetes: an overview of systematic reviews. Cochrane Database of Systematic Reviews 2015, Issue 7.

#### Excluded Cochrane overviews

Anderson L, Taylor RS. Cardiac rehabilitation for people with heart disease: an overview of Cochrane systematic reviews. Cochrane Database of Systematic Reviews 2014, Issue 12. *(≤2 reviews per group).*

Cates CJ, Oleszczuk M, Stovold E, Wieland LS. Safety of regular formoterol or salmeterol in children with asthma: an overview of Cochrane reviews. Cochrane Database of Systematic Reviews 2012, Issue 10. *(No control group).*

Cates CJ, Wieland LS, Oleszczuk M, Kew KM. Safety of regular formoterol or salmeterol in adults with asthma: an overview of Cochrane reviews. Cochrane Database of Systematic Reviews 2014, Issue 2. *(No control group).*

Derry CJ, Derry S, Moore RA. Sumatriptan (all routes of administration) for acute migraine attacks in adults - overview of Cochrane reviews. Cochrane Database of Systematic Reviews 2014, Issue 5. *(No control group).*

Flodgren G, Eccles MP, Shepperd S, Scott A, Parmelli E, Beyer FR. An overview of reviews evaluating the effectiveness of financial incentives in changing healthcare professional behaviours and patient outcomes. Cochrane Database of Systematic Reviews 2011, Issue 7. *(no DCA).*

Guay J, Choi P, Suresh S, Albert N, Kopp S, Pace NL. Neuraxial blockade for the prevention of postoperative mortality and major morbidity: an overview of Cochrane systematic reviews. Cochrane Database of Systematic Reviews 2014, Issue 1. *(≤2 reviews per group).*

Hindocha A, Beere L, Dias S, Watson A, Ahmad G. Adhesion prevention agents for gynaecological surgery: an overview of Cochrane reviews. Cochrane Database of Systematic Reviews 2015, Issue 1. *(No control group).*

Jones L, Othman M, Dowswell T, Alfirevic Z, Gates S, Newburn M, Jordan S, Lavender T, Neilson JP. Pain management for women in labour: an overview of systematic reviews. Cochrane Database of Systematic Reviews 2012, Issue 3. *(SD equal 0).*

Keus F, Gooszen HG, van Laarhoven CJHM. Open, small-incision, or laparoscopic cholecystectomy for patients with symptomatic cholecystolithiasis. An overview of Cochrane Hepato-Biliary Group reviews. Cochrane Database of Systematic Reviews 2010, Issue 1. *(No control group).*

O’Connell NE, Wand BM, McAuley J, Marston L, Moseley GL. Interventions for treating pain and disability in adults with complex regional pain syndrome- an overview of systematic reviews. Cochrane Database of Systematic Reviews 2013, Issue 4. *(≤2 reviews per group).*

Ryan R, Santesso N, Lowe D, Hill S, Grimshaw J, Prictor M, Kaufman C, Cowie G, Taylor M. Interventions to improve safe and effective medicines use by consumers: an overview of systematic reviews. Cochrane Database of Systematic Reviews 2014, Issue 4.

Singh JA, Wells GA, Christensen R, Tanjong Ghogomu E, Maxwell LJ, MacDonald JK, Filippini G, Skoetz N, Francis DK, Lopes LC, Guyatt GH, Schmitt J, La Mantia L, Weberschock T, Roos JF, Siebert H, Hershan S, Cameron C, Lunn MPT, Tugwell P, Buchbinder R. Adverse effects of biologics: a network meta-analysis and Cochrane overview. Cochrane Database of Systematic Reviews 2011, Issue 2. *(network meta-analysis).*

Welsh EJ, Evans DJ, Fowler SJ, Spencer S. Interventions for bronchiectasis: an overview of Cochrane systematic reviews. Cochrane Database of Systematic Reviews 2015, Issue 7. Art. *(SD equal 0).*

Wiffen PJ, Derry S, Moore RA, Aldington D, Cole P, Rice ASC, Lunn MPT, Hamunen K, Haanpaa M, Kalso EA. Antiepileptic drugs for neuropathic pain and fibromyalgia - an overview of Cochrane reviews. Cochrane Database of Systematic Reviews 2013, Issue 11. *(SD equal 0)*

#### Included non-Cochrane overviews

Ernst, E. and M.S. Lee, Acupuncture for rheumatic conditions: an overview of systematic reviews. Rheumatology (Oxford), 2010. 49(10): p. 1957–61.

Jacobs, W.C., et al., The evidence on surgical interventions for low back disorders, an overview of systematic reviews. Eur Spine J, 2013. 22(9): p. 1936–49.

Kitsiou, S., G. Pare, and M. Jaana, Effects of home telemonitoring interventions on patients with chronic heart failure: an overview of systematic reviews. J Med Internet Res, 2015. 17(3): p. e63.

Lauche, R., et al., A Systematic Overview of Reviews for Complementary and Alternative Therapies in the Treatment of the Fibromyalgia Syndrome. Evid Based Complement Alternat Med, 2015. 2015: p. 610615.

Lee, M.S., J.I. Kim, and E. Ernst, Is cupping an effective treatment? An overview of systematic reviews. J Acupunct Meridian Stud, 2011. 4(1): p. 1–4.

Lee, M.S., B. Oh, and E. Ernst, Qigong for healthcare: an overview of systematic reviews. JRSM Short Rep, 2011. 2(2): p. 7.

Lee, M.S. and E. Ernst, Systematic reviews of t’ai chi: an overview. Br J Sports Med, 2012. 46(10): p. 713–8.

Lee, M.S. and E. Ernst, Acupuncture for surgical conditions: an overview of systematic reviews. Int J Clin Pract, 2014. 68(6): p. 783–9.

#### Excluded non-Cochrane overviews (with reasons for exclusion)

Adams, L.V., et al., Interventions to improve delivery of isoniazid preventive therapy: an overview of systematic reviews. BMC Infect Dis, 2014. 14: p. 281. *(No control group).*

Andersen, J.H., et al., Risk factors for neck and upper extremity disorders among computers users and the effect of interventions: an overview of systematic reviews. PLoS One, 2011. 6(5): p. e19691. *(No control group).*

Bao, Y., et al., Complementary and alternative medicine for cancer pain: an overview of systematic reviews. Evid Based Complement Alternat Med, 2014. 2014: p. 170396. *(≤2 reviews per group).*

Berkhof, M., et al., Effective training strategies for teaching communication skills to physicians: an overview of systematic reviews. Patient Educ Couns, 2011. 84(2): p. 152–62. *(No control group).*

Boaz, A., et al., Effective implementation of research into practice: an overview of systematic reviews of the health literature. BMC Res Notes, 2011. 4: p. 212. *(No control group).*

Chan, R.J., E. Larsen, and *P. Chan*, Re-examining the evidence in radiation dermatitis management literature: an overview and a critical appraisal of systematic reviews. Int J Radiat Oncol Biol Phys, 2012. 84(3): p. e357–62. *(No control group).*

Cheung, A., et al., Overview of systematic reviews of the effectiveness of reminders in improving healthcare professional behavior. Syst Rev., 2012. 1: p. 36. *(≤2 reviews per group).*

Daka, Q. and V. Trkulja, Efficacy and tolerability of mono-compound topical treatments for reduction of intraocular pressure in patients with primary open angle glaucoma or ocular hypertension: an overview of reviews. Croat Med J, 2014. 55(5): p. 468–80. *(No control group).*

Ernst, E. and M.S. Lee, Acupressure: an overview of systematic reviews. J Pain Symptom Manage, 2010. 40(4): p. e3–7. *(≤2 reviews per group).*

Ernst, E., M.S. Lee, and T.Y. Choi, Acupuncture in obstetrics and gynecology: an overview of systematic reviews. Am J Chin Med, 2011. 39(3): p. 423–31. *(SD equal 0).*

Ernst, E., M.S. Lee, and T.Y. Choi, Acupuncture for insomnia? An overview of systematic reviews. Eur J Gen Pract, 2011. 17(2): p. 116–23. *(≤2 reviews per group).*

Ernst, E. and P. Posadzki, Complementary and alternative medicine for rheumatoid arthritis and osteoarthritis: an overview of systematic reviews. Curr Pain Headache Rep, 2011. 15(6): p. 431–7. *(≤2 reviews per group).*

Ernst, E., P. Posadzki, and M.S. Lee, Complementary and alternative medicine (CAM) for sexual dysfunction and erectile dysfunction in older men and women: an overview of systematic reviews. Maturitas, 2011. 70(1): p. 37–41. *(No control group).*

Faggion, C.M., Jr., et al., An overview of systematic reviews of the use of systemic antimicrobials for the treatment of periodontitis. Br Dent J, 2014. 217(8): p. 443–51. *(No control group).*

Foisy, M., et al., Overview of Reviews The prevention of eczema in infants and children: an overview of Cochrane and non-Cochrane reviews. Evid Based Child Health, 2011. 6(5): p. 1322–1339. *(No control group).*

Galvao, T.F., et al., Statins for early stage chronic kidney disease: an overview of reviews. Cardiovasc Hematol Disord Drug Targets, 2014. 14(3): p. 205–11. *(No control group).*

Gamble, J.M., et al., Incretin-based medications for type 2 diabetes: an overview of reviews. Diabetes Obes Metab, 2015. 17(7): p. 649–58. *(No control group).*

Gotink, R.A., et al., Standardized mindfulness-based interventions in healthcare: an overview of systematic reviews and meta-analyses of RCTs. PLoS One, 2015. 10(4): p. e0124344. *(No control group).*

Hagen, K.B., et al., Exercise therapy for bone and muscle health: an overview of systematic reviews. BMC Med, 2012. 10: p. 167. *(≤2 reviews per group).*

Heighes, P.T., et al., An overview of evidence from systematic reviews evaluating early enteral nutrition in critically ill patients: more convincing evidence is needed. Anaesth Intensive Care, 2010. 38(1): p. 167–74. *(No control group).*

Huguet, A., et al., Efficacy of psychological treatment for headaches: an overview of systematic reviews and analysis of potential modifiers of treatment efficacy. Clin J Pain, 2014. 30(4): p. 353–69. *(No control group).*

Hunt, K. and E. Ernst, The evidence-base for complementary medicine in children: a critical overview of systematic reviews. Arch Dis Child, 2011. 96(8): p. 769–76. *(Quality scores not reported).*

Iravani, M., et al., An overview of systematic reviews of normal labor and delivery management. Iran J Nurs Midwifery Res, 2015. 20(3): p. 293–303. *(No control group).*

Johal, A., et al., Mandibular advancement splint (MAS) therapy for obstructive sleep apnoea-an overview and quality assessment of systematic reviews. Sleep Breath, 2015. 19(3): p. 1101–8. *(No control group).*

Kang, H.S., et al., The use of acupuncture for managing gynaecologic conditions: An overview of systematic reviews. Maturitas, 2011. 68(4): p. 346–54. *(SD equal 0).*

Kansagara, D., et al., Transitions of Care from Hospital to Home: An Overview of Systematic Reviews and Recommendations for Improving Transitional Care in the Veterans Health Administration. 2015, Washington DC. *(No control group).*

Kim, T.H., et al., Dietary supplements for benign prostatic hyperplasia: an overview of systematic reviews. Maturitas, 2012. 73(3): p. 180–5. *(No control group).*

Kumar, A., S. Galeb, and B. Djulbegovic, Treatment of patients with multiple myeloma: an overview of systematic reviews. Acta Haematol, 2011. 125(1–2): p. 8–22. *(No control group).*

Laver, K., et al., Organising health care services for people with an acquired brain injury: an overview of systematic reviews and randomised controlled trials. BMC Health Serv Res, 2014. 14: p. 397. *(No control group).*

Lee, M.S., et al., Aromatherapy for health care: an overview of systematic reviews. Maturitas, 2012. 71(3): p. 257–60. *(SD equal 0).*

Lee, M.S., J.W. Kang, and E. Ernst, Does moxibustion work? An overview of systematic reviews. BMC Res Notes, 2010. 3: p. 284. *(SD equal 0).*

Lisy, K., H. White, and A. Pearson, Overview of reviews: mechanical interventions for the treatment and management of chronic obstructive pulmonary disease. Int J Nurs Pract, 2014. 20(6): p. 701–8. *(No control group).*

Liu, L., et al., Acupuncture for low back pain: an overview of systematic reviews. Evid Based Complement Alternat Med, 2015. 2015: p. 328196. *(≤2 reviews per group).*

Lockwood, C. and C. Stern, Interventions for the treatment of trachoma: an overview of Cochrane systematic reviews. Int J Nurs Pract, 2014. 20(6): p. 709–21. *(No control group).*

Lodge, C.J., et al., Overview of evidence in prevention and aetiology of food allergy: a review of systematic reviews. Int J Environ Res Public Health, 2013. 10(11): p. 5781–806. *(≤2 reviews per group).*

Long, L., et al., What is the clinical effectiveness and cost-effectiveness of conservative interventions for tendinopathy? An overview of systematic reviews of clinical effectiveness and systematic review of economic evaluations. Health Technol Assess, 2015. 19(8): p. 1–134. *(No control group).*

Lu, L.Y., G.Q. Zheng, and Y. Wang, An overview of systematic reviews of shenmai injection for healthcare. Evid Based Complement Alternat Med, 2014. 2014: p. 840650. *(No control group).*

Luo, J., et al., Traditional Chinese medicine injection for angina pectoris: an overview of systematic reviews. Am J Chin Med, 2014. 42(1): p. 37–59. *(Quality scores not reported).*

Luo, J., et al., Compound Danshen (Salvia miltiorrhiza) dripping pill for coronary heart disease: an overview of systematic reviews. Am J Chin Med, 2015. 43(1): p. 25–43. *(Quality scores not reported).*

Luo, J. and H. Xu, Outcome measures of chinese herbal medicine for coronary heart disease: an overview of systematic reviews. Evid Based Complement Alternat Med, 2012. 2012: p. 927392. *(Quality scores not reported).*

Luo, J., et al., Oral Chinese proprietary medicine for angina pectoris: an overview of systematic reviews/meta-analyses. Complement Ther Med, 2014. 22(4): p. 787–800. *(Quality scores not reported).*

Martineau, F., et al., Population-level interventions to reduce alcohol-related harm: an overview of systematic reviews. Prev Med, 2013. 57(4): p. 278–96. *(No control group).*

Mbuagbaw, L., et al., Mobile phone text messaging interventions for HIV and other chronic diseases: an overview of systematic reviews and framework for evidence transfer. BMC Health Serv Res, 2015. 15: p. 33. *(No control group).*

McCall, M.C., et al., Overview of systematic reviews: yoga as a therapeutic intervention for adults with acute and chronic health conditions. Evid Based Complement Alternat Med, 2013. 2013: p. 945895. *(No control group).*

Misfeldt, R., et al., Incentives for improving human resource outcomes in health care: overview of reviews. J Health Serv Res Policy, 2014. 19(1): p. 52–61. *(No control group).*

Momsen, A.M., et al., Multidisciplinary team care in rehabilitation: an overview of reviews. J Rehabil Med, 2012. 44(11): p. 901–12. *(No control group).*

Patel, N.N. and G.D. Angelini, Pharmacological strategies for the prevention of acute kidney injury following cardiac surgery: an overview of systematic reviews. Curr Pharm Des, 2014. 20(34): p. 5484–8. *(≤2 reviews per group).*

Plaszewski, M. and J. Bettany-Saltikov, Non-surgical interventions for adolescents with idiopathic scoliosis: an overview of systematic reviews. PLoS One, 2014. 9(10): p. e110254. *(SD equal 0).*

Posadzki, P., Is spinal manipulation effective for pain? An overview of systematic reviews. Pain Med, 2012. 13(6): p. 754–61. *(≤2 reviews per group).*

Posadzki, P., L. Watson, and E. Ernst, Herb-drug interactions: an overview of systematic reviews. Br J Clin Pharmacol, 2013. 75(3): p. 603–18. *(No control group).*

Posadzki, P., L.K. Watson, and E. Ernst, Adverse effects of herbal medicines: an overview of systematic reviews. Clin Med, 2013. 13(1): p. 7–12. *(No control group).*

Reif, K., U. de Vries, and F. Petermann, [What does really help against cancer-related fatigue? An overview of systematic reviews]. Pflege, 2012. 25(6): p. 439–57. *(No control group).*

Riech, A. and A. Schafer, [Standing- and gait therapy in adult patients after stroke: overview of reviews]. Rehabilitation (Stuttg), 2014. 53(6): p. 402–7. *(No control group).*

Rotta, I., et al., Effectiveness of clinical pharmacy services: an overview of systematic reviews (2000–2010). Int J Clin Pharm, 2015. *(No control group).*

Ryan, S.E., An overview of systematic reviews of adaptive seating interventions for children with cerebral palsy: where do we go from here? Disabil Rehabil Assist Technol, 2012. 7(2): p. 104–11. *(No control group).*

Sutcliffe, P., et al., Aspirin for prophylactic use in the primary prevention of cardiovascular disease and cancer: a systematic review and overview of reviews. Health Technol Assess, 2013. 17(43): p. 1–253. *(No control group).*

Terry, R., R. Perry, and E. Ernst, An overview of systematic reviews of complementary and alternative medicine for fibromyalgia. Clin Rheumatol, 2012. 31(1): p. 55–66. *(Quality scores not reported).*

Towler, P., A. Molassiotis, and S.G. Brearley, What is the evidence for the use of acupuncture as an intervention for symptom management in cancer supportive and palliative care: an integrative overview of reviews. Support Care Cancer, 2013. 21(10): p. 2913–23. *(No control group).*

Tricco, A.C., et al., Seeking effective interventions to treat complex wounds: an overview of systematic reviews. BMC Med, 2015. 13: p. 89. *(No control group).*

Wang, J. and X. Xiong, Outcome measures of chinese herbal medicine for hypertension: an overview of systematic reviews. Evid Based Complement Alternat Med, 2012. 2012: p. 697237. *(No control group).*

Wei, X., et al., Complementary and Alternative Medicine for the Management of Cervical Radiculopathy: An Overview of Systematic Reviews. Evid Based Complement Alternat Med, 2015. 2015: p. 793649. *(≤2 reviews per group).*

Wilson, M.G., et al., Counselling, case management and health promotion for people living with HIV/AIDS: an overview of systematic reviews. AIDS Behav, 2013. 17(5): p. 1612–25. *(≤2 reviews per group).*

Worswick, J., et al., Improving quality of care for persons with diabetes: an overview of systematic reviews - what does the evidence tell us? Syst Rev., 2013. 2: p. 26. *(No control group).*

Yang, C., et al., Efficacy and safety of acupuncture in children: an overview of systematic reviews. Pediatr Res, 2015. 78(2): p. 112–9. *(No control group).*

Young, B., et al., Preventing childhood falls within the home: overview of systematic reviews and a systematic review of primary studies. Accid Anal Prev, 2013. 60: p. 158–71. *(No control group).*

Young, T., et al., What are the effects of teaching evidence-based health care (EBHC)? Overview of systematic reviews. PLoS One, 2014. 9(1): p. e86706. *(No control group).*

Yuan, J., et al., The efficacy and safety of alpha-1 blockers for benign prostatic hyperplasia: an overview of 15 systematic reviews. Curr Med Res Opin, 2013. 29(3): p. 279–87. *(No control group).*

Zhang, J.H., D. Wang, and M. Liu, Overview of systematic reviews and meta-analyses of acupuncture for stroke. Neuroepidemiology, 2014. 42(1): p. 50–8. *(≤2 reviews per group).*

Zou, K., et al., Preventing childhood scalds within the home: Overview of systematic reviews and a systematic review of primary studies. Burns, 2015. 41(5): p. 907–24. *(No control group).*

Zwicker, J.G. and T.A. Mayson, Effectiveness of treadmill training in children with motor impairments: an overview of systematic reviews. Pediatr Phys Ther, 2010. 22(4): p. 361–77. *(No control group).*

## Abbreviations

AMSTAR: The Assessment of Multiple Systematic Reviews
CADTH: Canadian Agency for Drugs and Technologies in Health
CI: Confidence interval
DCA: Dual (co-)authorship
IQR: Interquartile ranges
MD: Mean difference
OQAQ: Overview Quality Assessment Questionnaire
SD: Standard deviation
SMD: Standardized mean difference
SRs: Systematic reviews
